# An international randomized phase III trial of pulse actinomycin-D versus multi-day methotrexate for the treatment of low risk gestational trophoblastic neoplasia; NRG/GOG 275

**DOI:** 10.1016/j.ygyno.2020.05.013

**Published:** 2020-05-24

**Authors:** Julian C. Schink, Virginia Filiaci, Helen Q. Huang, John Tidy, Matthew Winter, Jeanne Carter, Nancy Anderson, Katherine Moxley, Akira Yabuno, Sarah E. Taylor, Christina Kushnir, Neil Horowitz, David S. Miller

**Affiliations:** a Cancer Treatment Centers of America, Comprehensive Care and Research Center, Chicago, IL, USA; b NRG Oncology Statistics and Data Management Center, Roswell Park Comprehensive Cancer Center, Buffalo, NY, USA; c Sheffield Teaching Hospitals, NHS Trust, Royal Hallamshire Hospital, Glossop Rd, Sheffield S10 2JF, UK; d Memorial Sloan Kettering Cancer Center, 641 Lexington Avenue, New York, NY, USA; e Northwestern University, Chicago, IL, USA; f Oklahoma University Health Science Center, Oklahoma City, OK, USA; g Saitama Medical University International Medical Center, Saitama, Japan; h Gynecologic Oncology, Magee-Womens Hospital of University of Pittsburgh Medical Center, Pittsburgh, PA, USA; i Gynecologic Oncology, Women’s Cancer Center of Nevada, Las Vegas, NV, USA; j Harvard Medical School, Massachusetts General Hospital, Boston, MA, USA; k University of Texas Southwestern Medical Center, Dallas, TX, USA

**Keywords:** Actinomycin-D, Multi-day methotrexate, Gestational trophoblastic neoplasia, NRG, GOG

## Abstract

**Objectives.:**

Methotrexate and actinomycin-D are both effective first-line drugs for low-risk (WHO score 0–6) Gestational Trophoblastic Neoplasia (GTN) with considerable debate about which is more effective, less toxic, and better tolerated. The primary trial objective was to test if treatment with multi-day methotrexate (MTX) was inferior to pulse actinomycin-D (ACT-D). Secondary objectives included evaluation of severity and frequency of adverse events, and impact on quality of life (QOL).

**Methods.:**

This was a prospective international cooperative group randomized phase III two arm non-inferiority study (Clinical Trials Identifier: (NCT01535053). The control arm was ACT-D; the experimental arm was multi-day MTX regimen (institutional preference of 5 or 8 day). Outcome measures included complete response rate, recurrence rate, toxicity, and QOL as measured by FACT-G and FACIT supplemental items.

**Results.:**

The complete response rates for multi-day methotrexate and pulse actinomycin-D were 88% (23/26 patients) and 79% (22/28 patients) (p = NS) respectively, there were two recurrences in each arm, and 100% of patients survived. Significant toxicity was minimal, but mouth sores (mucositis), and eye pain were significantly more common in the MTX arm (p = 0.001 and 0.01 respectively). Quality of life showed no significant difference in overall quality of life, body image, sexual function, or treatment related side effects. The study was closed for low accrual rate (target 384, actual accrual 57), precluding statistical analysis of the primary objective.

**Conclusions.:**

The complete response rate for multi-day methotrexate was higher than actinomycin-D, but did not reach statistical significance. The multi-day MTX regimens were associated with significantly more mucositis and were significantly less convenient.

## 1. Introduction

The cure rate for low-risk GTN approaches 100%, primarily because of the inherent sensitivity of trophoblastic neoplasms to chemotherapy, the effective use of hCG as a marker of early disease, the identification of predictive factors of treatment response that has permitted individualization of therapy, and the use of combined surgery, radiation, and chemotherapy in the rare patients who fail initial chemotherapy [1–3]. Patients in this low-risk GTN group are those with Stage I disease (nonmetastatic) or Stage II and III disease (metastatic) with a risk score of <7. There are several different chemotherapy protocols for low-risk GTN that have produced comparable results in nonrandomized, retrospective studies. Taken together, these studies suggest that the 5-day methotrexate or the 8-day methotrexate–folinic acid protocol and the 5-day actinomycin D protocols or the bi-weekly single-dose actinomycin D protocols are significantly more effective than weekly intramuscular (IM) methotrexate. These studies also found that initial chemotherapy resistance is associated with older age, higher hCG, nonmolar antecedent pregnancy, histopathologic diagnosis of choriocarcinoma, presence of metastases, and a higher FIGO score. Common side effects of these regimens are stomatitis, mucositis of the gastrointestinal tract, pleuritis, conjunctivitis, and skin rash. Chemotherapy is continued until hCG values have returned to normal, and at least one course is administered after the first normal hCG level is achieved. A recent retrospective study compared relapse rates in women from the United Kingdom and the Netherlands with low-risk disease who were treated with methotrexate alternating with folinic acid [4]. A greater number of patients relapsed with only two consolidation courses than those who received three consolidation courses after reaching the institutional normal hCG value of <5 mIU/mL. Although there are differences in primary remission rates with each of the chemotherapy protocols, the cure rate remains high and most patients preserve their fertility.

While both methotrexate and Actinomycin-D are effective first-line drugs for low-risk (WHO score 0–6) gestational trophoblastic neoplasia there is lack of consensus as to a preferred regimen. When the predecessor to this trial, GOG-0174, a phase III randomized trial of bi-weekly IV actinomycin-D versus weekly IM methotrexate was initiated in 1999, the most commonly used North American regimen was low dose weekly IM methotrexate [5]. That Gynecologic Oncology Group trial demonstrated a significantly higher complete response rate for bi-weekly actinomycin-D regimen than for the weekly IM methotrexate regimen (complete response: 70% vs. 53%; p = 0.01) [5]. While the results of GOG-0174 were compelling evidence that pulse actinomycin-D is the more effective single day regimen, many Trophoblastic Disease Centers and others continue to use multi-day methotrexate regimens as their first line of therapy in the absence of a randomized controlled trial because of a perception of even higher complete response rates and lower toxicity compared with bi-weekly actinomycin-D. This prospective internationally designed study specifically addresses the persistent controversy over the relative toxicity and efficacy of these commonly used regimens and to investigate the impact of these treatments on overall QOL and treatment issues such as body image, sexual functioning, and patient reported side effects.

## 2. Materials and methods

### 2.1. Study design and objectives

**Fig. 1.**
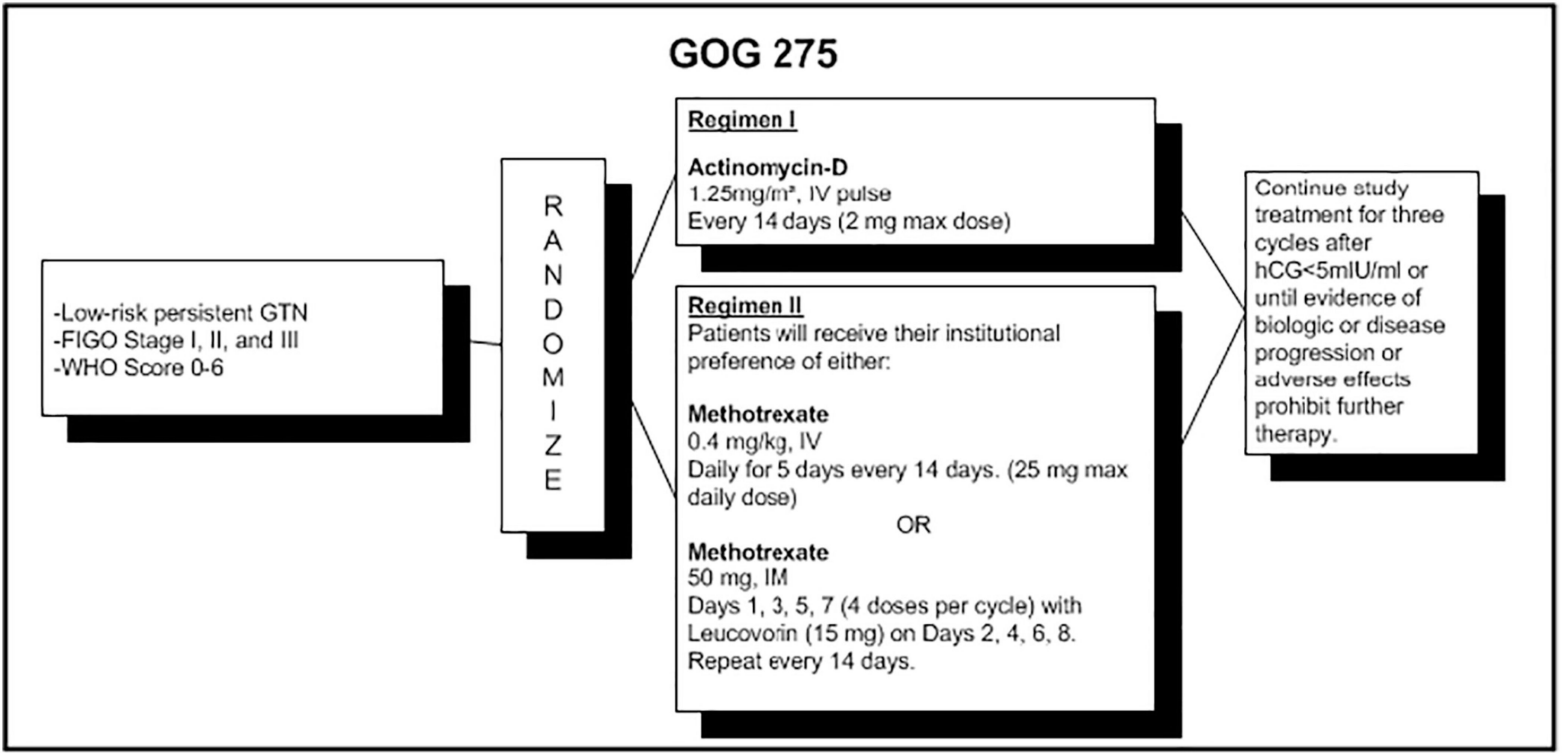
Study schema for NRG/GOG 275.

NRG/GOG 0275 was a cooperative group multicenter, two-arm, randomized phase 3 non-inferiority trial with a reference arm of actinomycin-D (ACT-D) and an experimental arm using one of two multi-day methotrexate (MTX) regimens chosen by the participating site. The study was approved by the Central IRB (CIRB) and each participating local IRB if required. Adult women with untreated F.I.G.O. Stage I, II, or III, low-risk, post-molar gestational trophoblastic neoplasia (GTN) or choriocarcinoma were eligible. The study schema is shown in Fig. 1. The primary endpoint was complete response defined as 3 consecutive bi-weekly values of serum human chorionic gonadotropin <5 over a minimum of 4 weeks with no values >5 mIU/ml. Secondary endpoints included the frequency and severity of adverse events in each arm, the frequency of post protocol therapeutic interventions, treatment arm differences in overall quality of life (QOL) measures and other patient reported outcomes, and the prognostic value of uterine artery pulsatility index. Treatment emergent adverse events for this study were graded and categorized using CTCAE v4. All patients signed written informed consent according to institutional and federal guidelines before enrollment.

### 2.2. Quality of life

The QOL and Patient-reported outcomes (PRO) instruments included: FACT-G (27 items) – for QOL endpoint (primary QOL objective); FACIT supplemental items (11 items) – for treatment-related side effects (exploratory); and exploratory items for treatment-related disruption and inconvenience (5 items) [6,7].

International participation in this study began following translation of QOL instruments into Korean and Japanese languages. PRO/QOL assessments were scheduled at the following time points:

1. Prior to cycle 1 (baseline).
2. Prior to cycle 3 (4 weeks after cycle 1 if off study treatment prior to cycle 3).
3. Prior to cycle 5 (8 weeks after cycle 1 if off study treatment prior to cycle 5).
4. Prior to cycle 7 (12 weeks after cycle 1 if off study treatment prior to cycle 7).
5. 26 weeks after starting study treatment.

The Functional Assessment of Cancer Therapy - General (FACT-G) is a scale for assessing general QOL of patients with any types of cancer. It consists of four subscales: Physical Well-Being (7 items), Functional Well-Being (7 items), Social/Family Well-Being (7 items), and Emotional Well-Being (6 items). Each item in the FACT-G is scaled using a 5-point scale (0 = not at all; 1 = a little bit; 2 = somewhat; 3 = quite a bit; 4 = very much) [7]. For the negative statements (or questions), reversal was performed prior to score calculation. According to the FACIT measurement system, a subscale score was the summation of the individual item scores if >50% of subscale items were answered. When unanswered items existed, a subscale score was prorated by multiplying the mean of the answered item scores by the number of items in the subscale. The total FACT-G score was calculated as the sum of the subscale scores if >80% of the FACT-G items provide valid answers and all of the component subscales have valid scores [6]. The FACT-G total score ranged from 0 to 108 and a higher score indicates better QOL. The minimal important difference (MID) of the FACT-G was 3–7 points.

The eleven FACIT supplemental items were selected from the FACT disease specific subscales addressing body image (4 items), sexual function (3 items), and treatment-related side effects (4 items). The scoring for these supplemental items followed the same method for the subscales of the FACT-G.

The treatment concerns were measured with four items addressing the factors associated with chemotherapy. Each item is scaled using the same 5-point scale (0 = not at all; 1 = a little bit; 2 = somewhat; 3 = quite a bit; 4 = very much) as used for the FACIT items. A score 2 or more is considered ‘somewhat or more’ bothered or disruptive by the treatment. A total score was calculated using the same method for FACT-G subscales.

The overall degree of life disruption from the study treatment was evaluated with a single item which is scaled from 0 = no disruption to 10 = extremely disruptive to my life.

### 2.3. Study population

Eligible patients had F.I.G.O. Stage I, II, or III post molar GTN or choriocarcinoma. Patients could have had a second curettage but must still meet GTN criteria after the curettage. Post molar GTN patients must have undergone evacuation of a complete or partial hydatidiform mole and then meet the criteria for GTN (see Table 1); and W.H.O. risk score 0–6.

### 2.4. Treatments

Regimen 1: IV pulse actinomycin-D (1.25 mg/m^2^) once every 14 days with a maximum dose of 2 mg.

Regimen 2: Institutional preference of either IV methotrexate (0.4 mg/kg) daily for 5 days every 14 days with a maximum daily dose of 25 mg.

OR

IM methotrexate (50 mg) on Days 1,3,5,7 (4 doses per cycle) with leucovorin (15 mg) orally on Days 2, 4, 6, 8 within 24–30 h of the preceding day’s injection. This is repeated every 14 days.

### 2.5. Statistical analysis

The design was to provide a direct assessment of the null hypothesis that the efficacy attributed to multi-day methotrexate regimens is inferior to that of ACT-D given every two weeks. In the event that the null hypothesis of inferiority was rejected, a test of the superiority of the methotrexate regimen would be carried out and reported. The study was designed to have 80% power to detect an increase in response rate of 11% if the true response rate in the reference arm was 70%. The QOL hypotheses were secondary objectives, therefore, they were not included in the statistical testing strategy or sample size calculation for the primary clinical objective which focused on complete response. Since the study accrual was closed prematurely the primary objective could not be analyzed and the analysis on QOL measures were considered exploratory and only 95% confidence intervals were provided for estimated treatment differences. Patients who completed baseline and at least one follow-up QOL assessments were considered evaluable for QOL measures. A linear mixed model was used to estimate the treatment differences in patient-reported QOL scores adjusting for patient’s pretreatment score, treatment assignment, and age at enrollment and stratified by the country where treatment was given. Missing at random was assumed for missing values since the primary reasons for missing an assessment was administrative error (e.g. not administrating the questionnaire to patients). Patients were classified by their randomly assigned regimen rather than the treatment received. The assessment time points were treated as categorical since they are not equally spaced. The covariance matrix among the repeated QOL scores reported by the same patient is assumed to be unstructured. To reflect the observed covariance pattern of the QOL scores, the ‘empirical’ variance was used in estimating the precision of parameter estimates. First, the interactions between assessment time points and treatment assignments were tested at a significant level of 0.05 for the constant differential effects of treatments over time. If the interaction effect was not statistically significant, an overall treatment effect was estimated by a weighted average of estimates from each time point. If the testing for interaction was rejected treatment comparison was performed for each assessment time.

All the analyses in this report are exploratory since the study was closed prematurely. All analyses were undertaken using SAS/STAT Software 9.4.

## 3. Results

### 3.1. Patient accrual

**Fig. 2.**
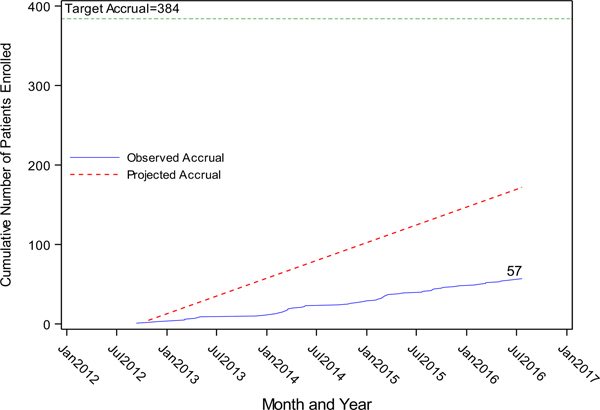
Cumulative accrual.

**Fig. 3.**
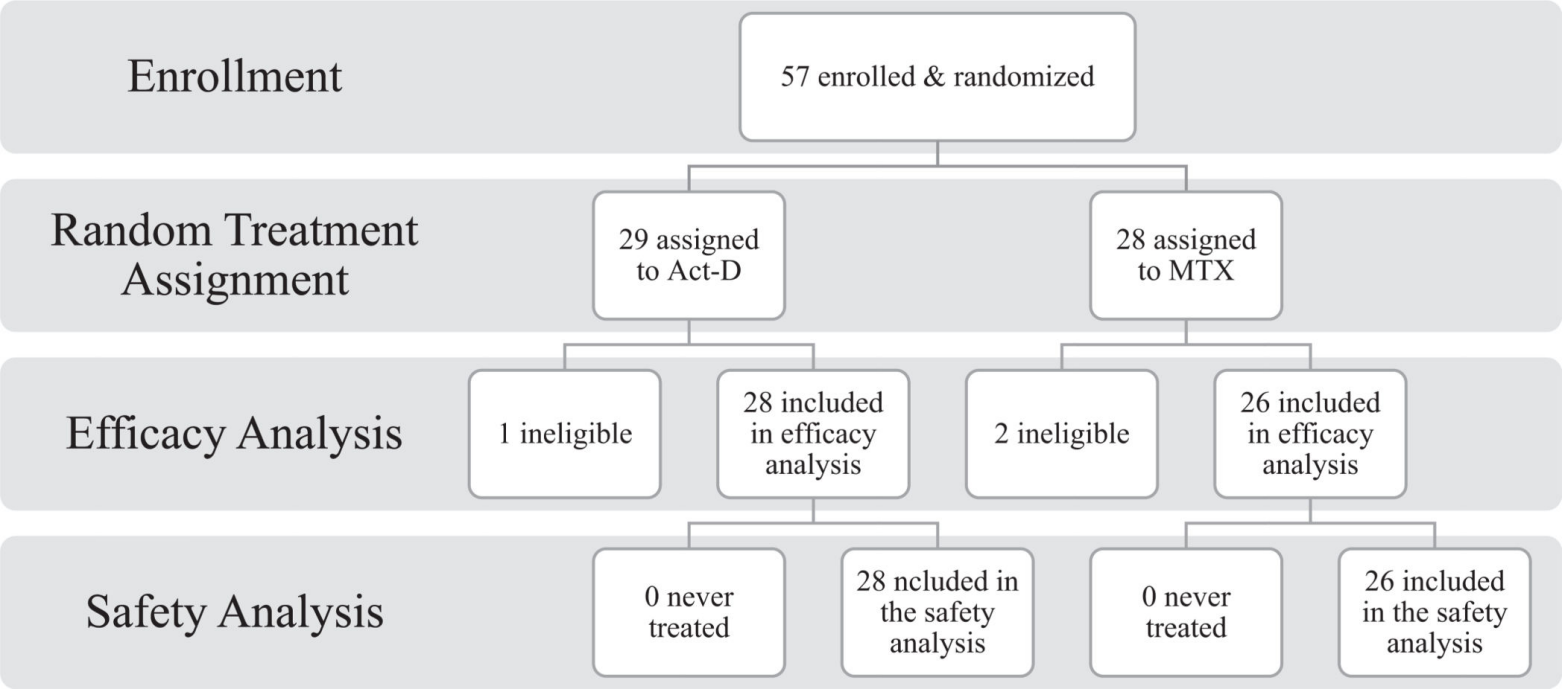
Consort diagram.

The study was opened to accrual on 06/18/2012. The first participant was enrolled on September 11, 2012. The study was closed on 9/20/2016 due to slow accrual, despite a concerted international effort to enhance accrual (Fig. 2); and does not meet the protocol-specified statistical requirements for interim or final analysis. International participation included multiple GOG member institutions in Korea, Japan, and Sheffield in the United Kingdom. A total of 57 patients (15% of the study goal) were enrolled and received a random treatment assignment. (See Consort diagram, Fig. 3) As of October 9, 2018, the median follow-up time is 24.2 months.

### 3.2. Patient and tumor characteristics

Among the 57 participants, 3 were deemed ineligible on central eligibility review. Of all eligible patients enrolled, 1 has withdrawn consent to be followed prior to definitive response evaluation. Patient and tumor characteristics of all eligible enrolled patients are presented in Supplemental Table 1. Complete moles were diagnosed in 38 patients and choriocarcinoma was diagnosed in only 5. The median WHO score at study entry is 2.5.

### 3.3. Adverse events

All reported adverse events, categorized using CTCAE v4 are included in Supplemental Table 2.

Grade 3 adverse event were reported in 16 participants, 10 in the MTX arm and 6 in the ACT-D arm. No grade 4 adverse events were reported. The most common grade 3 toxicity in the MTX arm was mucositis occurring in 4 of 26 evaluable patients. The other grade 3 toxicities reported for MTX patients and ACT-D patients were sparsely distributed across the toxicity spectrum and were reported in 1 or 2 patients per arm. Grade 1 alopecia, defined as “hair loss of <50% of normal for that individual that is not obvious from a distance…” was reported in 7 of 28 ACT-D patients (25%); and 4 of 26 MTX patients, with 1 MTX patient reporting grade 2 alopecia (19%). Considering all grades of toxicity reveals that mouth sores (mucositis), and eye disorders (mostly related to watering and dry eye) were significantly more common in the MTX arm (p = 0.001 and 0.01 respectively).

### 3.4. Treatment outcomes

At the time of analysis all patients in this study are alive and 79% and 88% met the criteria for a complete response on the actinomycin-D and methotrexate arms respectively (Table 2). Two patients in each arm subsequently developed recurrence and are alive after additional treatment. The median number of cycles for a CR was 6 in both the Act-D arm and the 5-d MTX group, and 7 in the 8 day MTX group (Table 3) An insufficient number of patients had the optional uterine artery pulsatility index evaluation to evaluate its prognostic value.

### 3.5. Quality of life and patient-reported outcomes

A total of 54 patients (n = 27 for each group) provided evaluable QOL/PRO assessments. The FACT-G score reported at baseline was 82.3 by patients on ACT-D and 81.9 by those on MTX. After adjustment for patient’s age, baseline score, and the country the treatment was administered, the changes of the FACT-G score over assessment time were not significantly different between the two treatment groups (p-value = 0.76 for the interaction between time and treatment groups). The patients on MTX reported 0.95 (95% CI: −4.7–6.6) points higher on average across the assessment time when compared to those on ACT-D (Supplemental Fig. 1).

The FACIT supplemental items were grouped into three subscales to evaluate treatment effects on body image, sexual function, treatment related side effects. After adjustment for patient’s age, baseline score, and the country the treatment was administered, the changes of the FACIT supplemental items score over assessment time were not significantly different between the two treatment groups (p = 0.9 for body image, p = 0.1 for side effects, and p = 0.6 for sexual function, for the interaction). On average across the assessment time the patients on MTX reported 1.1 points higher (95% CI: 0.09–2.05) on body image, 1.4 points higher (95% CI: 0.4–2.4) on sexual function, and 0.04 points lower (95% CI: −1.1–1.0) on treatment related side effect, when compared to those on ACT-D (Supplemental Fig. 2).

Treatment concerns data was collected on patients who were on study treatment at the time the assessment was done. A majority of the patients were off treatment at 26 weeks and only 8 patients (4 on each arm) provided treatment concerns and disruption data at that assessment. Therefore, treatment concerns and disruption were summarized only for assessments received at 4, 8, and 12 weeks after starting study treatment.

The treatment issues that were ‘somewhat’ or more bothersome or disruptive to patients are presented in Supplemental Fig. 3. Both treatment methods were disruptive or bothersome to patients but in different aspects. For example, initially 36% of ACT-D patients found the amount of time to have a treatment bothersome compared with 21% of methotrexate patients. While as many as 58% of methotrexate patients, at week 8, were bothered by the number of treatments needed per week in contrast with only 15% of ACT_D patients sharing this complaint.

## 4. Discussion

### 4.1. Treatment response

In low risk GTN the upfront complete response rate is an important endpoint because approximately 25% of women failing to achieve complete response with their initial chemotherapy regimen will ultimately require surgery and/or multi-agent etoposide based chemotherapy [8,9]. In GOG-0174, which was a study of weekly MTX versus pulse actinomycin-D in low risk GTN, the complete response rate for pulse actinomycin-D was 70%, significantly better than weekly methotrexate with only a 53% complete response rate (p = 0.01) [5]. In the current study the complete response rate for pulse actinomycin-D was higher at 79% despite a very similar patient population but smaller sample size. Lurain et al. described the toxicity and efficacy of the 5 day methotrexate regimen in a comparable population noting 4.7% of patients experienced toxicity requiring a change from methotrexate to actinomycin-D and an 89.3% (226/253) complete remission rate when treated with single-agent methotrexate (0.4 mg/kg IV push q.d. × 5d q.o. week) [8]. Berkowitz described the New England Trophoblastic Disease Center experience with the 8 day methotrexate with folinic acid rescue regimen with a 90% complete response rate [9]. A Cochrane review concluded that the 8 day regimen did not offer an advantage in efficacy or adverse event profile over the 5 day regimen [10]. In the current study the complete response rate for multi-day MTX reproduced these prior retrospective study results with an 88% CR, which was higher than Act-D but not statistically significant [12].

### 4.2. Toxicity and patient reported outcomes

MTX regimens were significantly more likely to cause mouth and eye toxicity (mucositis and watering and dry eye disorders) than Act-D. The MTX regimens were also significant less convenient as measured in the QOL evaluation. Alopecia was uncommon and not identified as an issue in either the toxicity measurement or QOL evaluation (specifically “body image”) in either arm of the study. While alopecia is often cited as a reason not to use Act-D for GTN patients, that risk is not supported by this study [11]. Vascular access concerns were not an issue, with only one case of grade 2 phlebitis in the actinomycin-D arm and none in the methotrexate arm. Both study regimens were remarkably well tolerated with no patients discontinuing their treatment regimen because of toxicity. Historically there is a perception that Act-D is a more toxic regimen, this was not the case in this study, the only significantly more common toxicities occurred in the MTX arm, specifically mucositis and eye disorders.

### 4.3. Accrual challenges

Accrual to this phase III study comparing treatment regimens that have strong institutional bias as well as logistic barriers was disappointing and lead to study closure prior to meeting its accrual goal thus preventing statistical analysis of the primary endpoint of complete response. Specifically, many of the busiest trophoblastic disease centers were unable or unwilling to accrue patients to this study. The logistical challenges of a multiday regimen further inhibited accrual. The two different multiday methotrexate regimens are a design weakness of this trial. This use of two regimens was done because the treatments are relatively comparable in dose intensity, and decades long institutional preferences were inflexible for logistic reasons. The five-day methotrexate regimen is an intravenous treatment generally administered by a chemotherapy certified nurse Monday through Friday in an infusion center. The eight-day regimen avoids the expertise of intravenous access by administration as an intramuscular injection given every other day; this requires a weekend encounter which is not practical in some sites. While conducting a three-armed study might be scientifically desirable it was not statistically feasible. Despite studying the same population as GOG 174, which accrued 240 patients in 7.5 years (32 patients per year), this study accessioned patients at only 15 patients per year. The addition of international participation in four foreign countries helped but did not solve the slow accrual problem. Even broader international participation, including at dedicated trophoblastic disease centers, was prevented by numerous logistical and regulatory barriers. These barriers must be addressed by the international scientific community if we are to succeed in studying rare tumors.

## 5. Conclusions

While the multiday methotrexate regimens demonstrated a slightly higher complete response rate this difference was not significant. This study is the first phase III study of these regimens addressing quality of life and patient reported outcomes. Both methotrexate and Actinomycin-D regimens are remarkably well tolerated with QOL and patient reported outcomes showing no significant difference between arms in overall quality of life, body image, sexual function, or overall treatment related side effects. However, the multiday methotrexate regimens were significantly more likely to cause mucositis and eye disorders; and they were perceived by patients as much more bothersome with respect to the frequency of treatments needed.

## Supplementary Material

MMC1

## Figures and Tables

**Table 1:** Criteria for GTN.

Post molar GTN was defined as:
<10% decrease in the hCG level using as a reference the first value in the series of 4 values taken over a period of 3 weeks (>50 mIU/ml minimum).
OR
>20% sustained rise in the hCG taking as a reference the first value in the series of 3 values taken over a period of 2 weeks (>50 mIU/ml minimum).
OR
persistently elevated hCG level a period of 6 months or more following the initial curettage (>50 mIU/ml minimum).
OR
Sheffield criteria: hCG level >20,000 four weeks after evacuation of molar pregnancy.

**Table 2:** Responses by regimen. GOG-0275 disease outcome. Data as of 10/09/18.

Outcome	Actinomycin-D		Methotrexate		Total
	N	%	N	%	N
Best hCG response					
Data missing	1	3.6	0	0	1
Complete response (CR)	22	78.6	23	88.5	45
Treatment failure	4	14.3	2	7.7	6
Treatment failure not documented	1	3.6	1	3.8	2
Recurrence post CR	2	9.1	2	8.7	4
Survival status					
Alive	28	100.0	26	100.0	54

**Table 3:** Chemotherapy cycles summary by agent and route.

		Regimen		
		Actinomycin-D	Methotrexate	
		Agent/route	Agent/route	
		IV pulse	IM	IV
		actinomycin-D	methotrexate	methotrexate
Number of cycles	Minimum	2.0	4.0	3.0
	Median	6.0	7.0	6.0
	Maximum	16.0	10.0	16.0

## H I G H L I G H T S

- Multi-day methotrexate had an 88% complete response rate in low risk GTN.
- Every two week actinomycin-D had a 79% complete response rate in low risk GTN.
- The survival rate for patients in this study was 100%
- Quality of life showed no significant difference in overall quality of life, body image, or sexual function.
- The MTX arm reported more treatment concerns and disruption of lifestyle, but this was not significant.
