# Supplementary material for: An international randomized phase III trial of pulse actinomycin-D versus multi-day methotrexate for the treatment of low risk gestational trophoblastic neoplasia; NRG/GOG 275
Source: Gynecol Oncol. Author manuscript; Available in PMC 2020 Aug 18. (PMC7432963; doi:10.1016/j.ygyno.2020.05.013)
Supplement: MMC1 [file NIHMS1598475-supplement-MMC1.docx]

**Supplement Figure 1**

Caption: Supplemental Figure 1. The plot lines present the patient-reported FACT-G scores.

The least-squares means estimates were obtained from a fitted mixed model adjusting for pre-treatment score (baseline score), patient’s age at the enrollment, and the country the treatment was administered. A larger score indicates favorable or better QOL. The least squares means differences were estimated from the fitted mixed models.


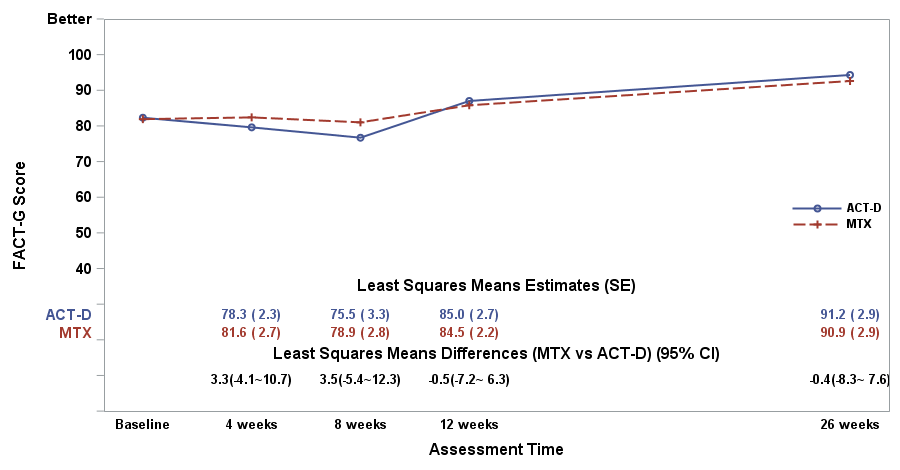


**Supplemental Figure 2.**


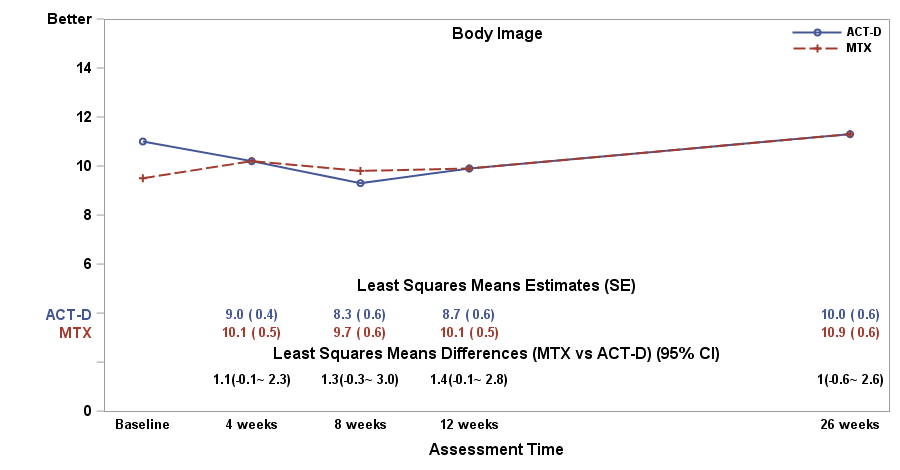


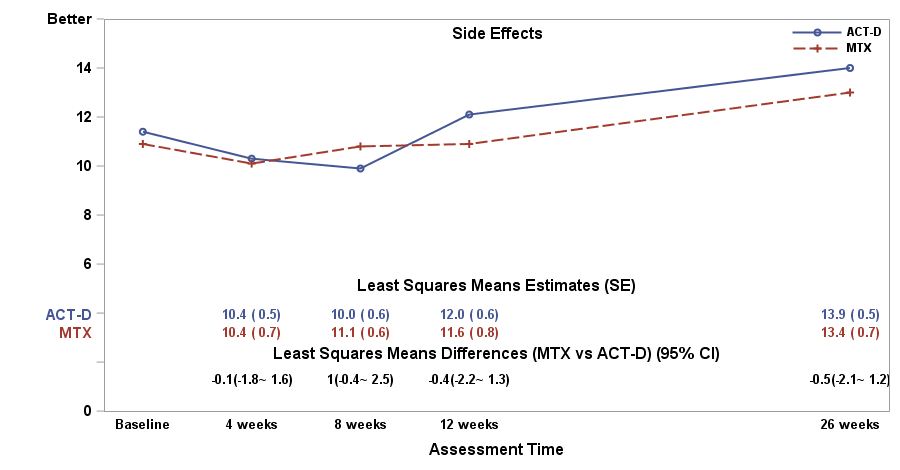


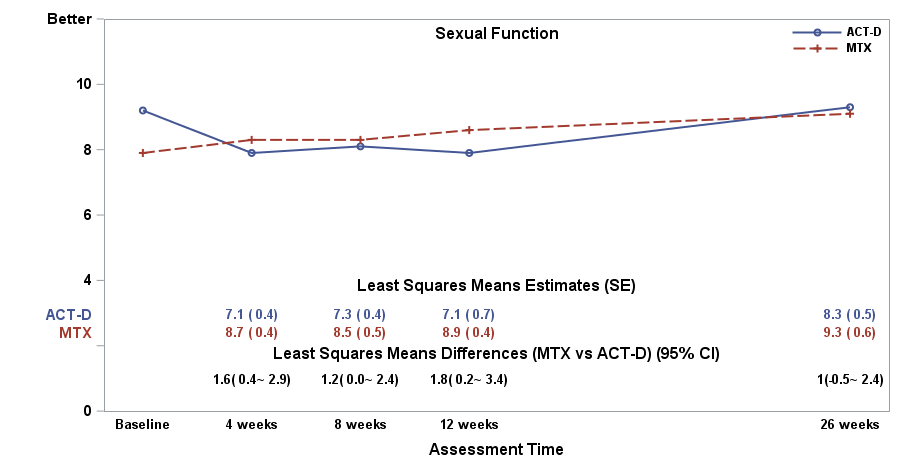


Caption: supplemental Figure 2. The plot lines present the patient-reported body image (3A), side effects (3B), and sexual function subscale scores (3C). The least-squares means estimates were obtained from a fitted mixed model adjusting for pre-treatment score (baseline score), patient’s age at the enrollment, and the country the treatment was administered. A larger score indicates favorable or better QOL or few side effects. The least squares means differences were estimated from the fitted mixed models.

Supplemental Figure 3

**Supplemental Figure 3.**


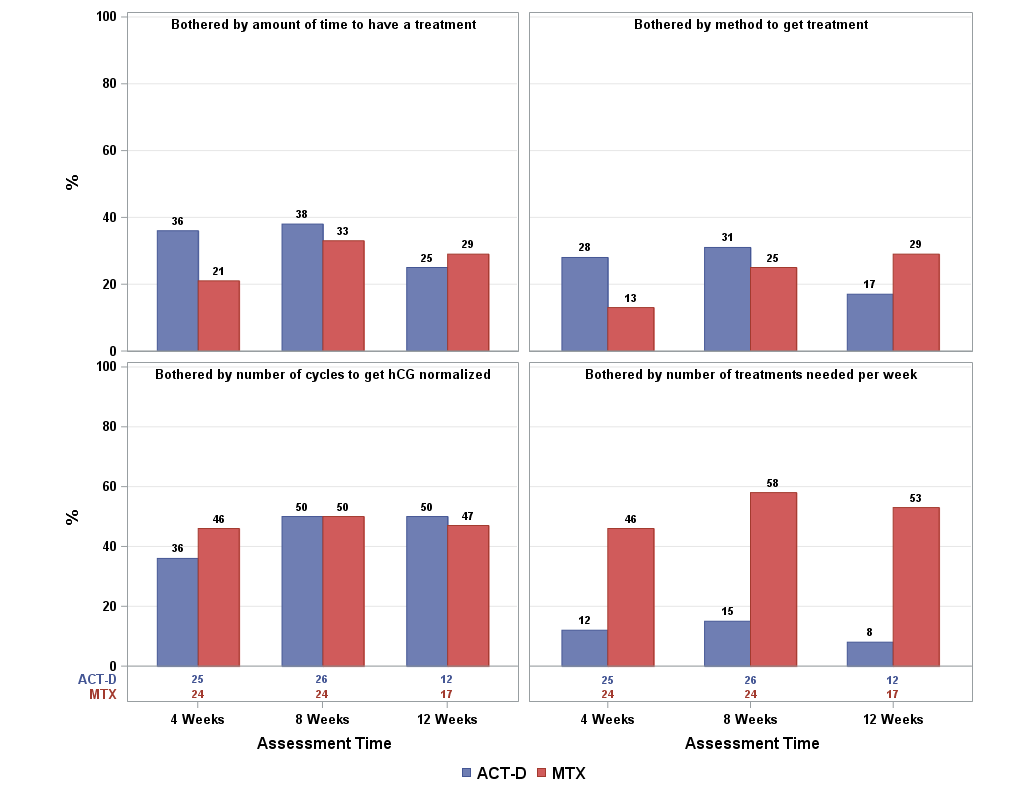


Caption: Percentage of patients reporting being ‘somewhat’ or more bothersome or disruptive by treatment issues. The numbers at the bottom of bars are the number of patients providing treatment concerns and disruption data in each treatment arms.

# Supplemental Table 1

# Caption: GOG-­0275 Patient and Tumor Characteristics for All Eligible Enrolled Patients

| **Characteristic** | **Regimen** | | | | **Total** | |
| --- | --- | --- | --- | --- | --- | --- |
|  | **Actinomycin-D** | | **Methotrexate** | |  |  |
|  | **N** | **%** | **N** | **%** | **N** | **%** |
| Age Group |  |  |  |  |  |  |
| 10-19 | 1 | 3.6 | 1 | 3.8 | 2 | 3.7 |
| 20-29 | 14 | 50.0 | 11 | 42.3 | 25 | 46.3 |
| 30-39 | 11 | 39.3 | 11 | 42.3 | 22 | 40.7 |
| 40-49 | 2 | 7.1 | 3 | 11.5 | 5 | 9.3 |
| Ethnicity |  |  |  |  |  |  |
| Hispanic or Latino | 2 | 7.1 | 2 | 7.7 | 4 | 7.4 |
| Non-Hispanic | 26 | 92.9 | 24 | 92.3 | 50 | 92.6 |
| Race |  |  |  |  |  |  |
| Asian | 6 | 21.4 | 6 | 23.1 | 12 | 22.2 |
| Black/African American | 6 | 21.4 | 1 | 3.8 | 7 | 13.0 |
| White | 16 | 57.1 | 19 | 73.1 | 35 | 64.8 |
| Molar Class |  |  |  |  |  |  |
| Missing | 0 | 0 | 1 | 3.8 | 1 | 1.9 |
| Complete mole | 21 | 75.0 | 17 | 65.4 | 38 | 70.4 |
| Choriocarcinoma | 1 | 3.6 | 4 | 15.4 | 5 | 9.3 |
| Partial Mole | 6 | 21.4 | 4 | 15.4 | 10 | 18.5 |
| WHO Score |  |  |  |  |  |  |
| Missing | 1 | 3.6 | 0 | 0 | 1 | 1.9 |
| 0 | 5 | 17.9 | 2 | 7.7 | 7 | 13.0 |
| 1 | 5 | 17.9 | 5 | 19.2 | 10 | 18.5 |
| 2 | 4 | 14.3 | 6 | 23.1 | 10 | 18.5 |
| 3 | 5 | 17.9 | 4 | 15.4 | 9 | 16.7 |
| 4 | 4 | 14.3 | 3 | 11.5 | 7 | 13.0 |
| 5 | 2 | 7.1 | 4 | 15.4 | 6 | 11.1 |
| 6 | 2 | 7.1 | 2 | 7.7 | 4 | 7.4 |
| Registration beta hCG |  |  |  |  |  |  |
| 10.1-100.0 | 2 | 7.1 | 0 | 0 | 2 | 3.7 |
| 100.1-1500.0 | 8 | 28.6 | 2 | 7.7 | 10 | 18.5 |
| 1500.1-5000.0 | 4 | 14.3 | 7 | 26.9 | 11 | 20.4 |
| 5000.1-10000.0 | 2 | 7.1 | 4 | 15.4 | 6 | 11.1 |
| 10000.1-100000.0 | 11 | 39.3 | 11 | 42.3 | 22 | 40.7 |
| 100000.1-1000000.0 | 1 | 3.6 | 2 | 7.7 | 3 | 5.6 |
| Strata: Country |  |  |  |  |  |  |
| Canada | 1 | 3.6 | 3 | 11.5 | 4 | 7.4 |
| Japan | 3 | 10.7 | 3 | 11.5 | 6 | 11.1 |
| Korea | 2 | 7.1 | 1 | 3.8 | 3 | 5.6 |
| UK | 3 | 10.7 | 5 | 19.2 | 8 | 14.8 |
| US | 19 | 67.9 | 14 | 53.8 | 33 | 61.1 |
| Strata: Methotrexate Regimen |  |  |  |  |  |  |
| IM | 11 | 39.3 | 9 | 34.6 | 20 | 37.0 |
| IV | 17 | 60.7 | 17 | 65.4 | 34 | 63.0 |
| **Total** | 28 | 51.9 | 26 | 48.1 | 54 | 100.0 |

**Supplemental Table 2: Adverse Events**

**Adverse events were graded with CTCAE version 4**

# Distribution of GOG-0275 Patients by Highest Grade Adverse Event

By System Organ Class for All Reported Adverse Events without Regard to Attribution

Data as of 10/09/18

|  | **Actinomycin-D (n=28)** | | | | | **Methotrexate (n=26)** | | | | |
| --- | --- | --- | --- | --- | --- | --- | --- | --- | --- | --- |
|  | **No. and (%) of Patients by Grade** | | | | | **No. and (%) of Patients by Grade** | | | | |
|  | **1** | **2** | **3** | **4** | **5** | **1** | **2** | **3** | **4** | **5** |
| **System Organ Class** |  | | | | | | | | | |
| Overall Highest Grade | 7 | 14 | 6 | 0 | 0 | 4 | 10 | 10 | 0 | 0 |
|  | (25.0) | (50.0) | (21.4) | (0.0) | (0.0) | (15.4) | (38.5) | (38.5) | (0.0) | (0.0) |
| Blood and Lymphatic System Disorders | 9 | 3 | 1 | 0 | 0 | 12 | 2 | 2 | 0 | 0 |
|  | (32.1) | (10.7) | (3.6) | (0.0) | (0.0) | (46.2) | (7.7) | (7.7) | (0.0) | (0.0) |
| Cardiac Disorders | 1 | 0 | 0 | 0 | 0 | 1 | 0 | 0 | 0 | 0 |
|  | (3.6) | (0.0) | (0.0) | (0.0) | (0.0) | (3.8) | (0.0) | (0.0) | (0.0) | (0.0) |
| Ear and Labyrinth Disorders | 3 | 0 | 0 | 0 | 0 | 4 | 0 | 0 | 0 | 0 |
|  | (10.7) | (0.0) | (0.0) | (0.0) | (0.0) | (15.4) | (0.0) | (0.0) | (0.0) | (0.0) |
| Eye Disorders | 5 | 0 | 0 | 0 | 0 | 11 | 2 | 0 | 0 | 0 |
|  | (17.9) | (0.0) | (0.0) | (0.0) | (0.0) | (42.3) | (7.7) | (0.0) | (0.0) | (0.0) |
| Gastrointestinal Disorders | 14 | 10 | 1 | 0 | 0 | 9 | 7 | 5 | 0 | 0 |
|  | (50.0) | (35.7) | (3.6) | (0.0) | (0.0) | (34.6) | (26.9) | (19.2) | (0.0) | (0.0) |
| General Disorders and Administration Site Conditions | 17 | 3 | 1 | 0 | 0 | 13 | 5 | 0 | 0 | 0 |
|  | (60.7) | (10.7) | (3.6) | (0.0) | (0.0) | (50.0) | (19.2) | (0.0) | (0.0) | (0.0) |
| Immune System Disorders | 0 | 0 | 0 | 0 | 0 | 1 | 0 | 0 | 0 | 0 |
|  | (0.0) | (0.0) | (0.0) | (0.0) | (0.0) | (3.8) | (0.0) | (0.0) | (0.0) | (0.0) |
| Infections and Infestations | 3 | 1 | 1 | 0 | 0 | 0 | 6 | 1 | 0 | 0 |
|  | (10.7) | (3.6) | (3.6) | (0.0) | (0.0) | (0.0) | (23.1) | (3.8) | (0.0) | (0.0) |
| Injury, Poisoning and Procedural Complications | 1 | 0 | 0 | 0 | 0 | 0 | 0 | 0 | 0 | 0 |
|  | (3.6) | (0.0) | (0.0) | (0.0) | (0.0) | (0.0) | (0.0) | (0.0) | (0.0) | (0.0) |
| Investigations | 6 | 2 | 2 | 0 | 0 | 5 | 4 | 2 | 0 | 0 |
|  | (21.4) | (7.1) | (7.1) | (0.0) | (0.0) | (19.2) | (15.4) | (7.7) | (0.0) | (0.0) |
| Metabolism and Nutrition Disorders | 7 | 1 | 0 | 0 | 0 | 6 | 3 | 1 | 0 | 0 |
|  | (25.0) | (3.6) | (0.0) | (0.0) | (0.0) | (23.1) | (11.5) | (3.8) | (0.0) | (0.0) |
| Musculoskeletal and Connective Tissue Disorders | 5 | 1 | 0 | 0 | 0 | 4 | 0 | 0 | 0 | 0 |
|  | (17.9) | (3.6) | (0.0) | (0.0) | (0.0) | (15.4) | (0.0) | (0.0) | (0.0) | (0.0) |
| Nervous System Disorders | 8 | 0 | 1 | 0 | 0 | 4 | 1 | 0 | 0 | 0 |
|  | (28.6) | (0.0) | (3.6) | (0.0) | (0.0) | (15.4) | (3.8) | (0.0) | (0.0) | (0.0) |
| Psychiatric Disorders | 4 | 0 | 1 | 0 | 0 | 7 | 1 | 0 | 0 | 0 |
|  | (14.3) | (0.0) | (3.6) | (0.0) | (0.0) | (26.9) | (3.8) | (0.0) | (0.0) | (0.0) |
| Renal and Urinary Disorders | 2 | 0 | 0 | 0 | 0 | 3 | 0 | 0 | 0 | 0 |
|  | (7.1) | (0.0) | (0.0) | (0.0) | (0.0) | (11.5) | (0.0) | (0.0) | (0.0) | (0.0) |
| Reproductive System and Breast Disorders | 9 | 5 | 0 | 0 | 0 | 11 | 2 | 0 | 0 | 0 |
|  | (32.1) | (17.9) | (0.0) | (0.0) | (0.0) | (42.3) | (7.7) | (0.0) | (0.0) | (0.0) |
| Respiratory, Thoracic and Mediastinal Disorders | 7 | 0 | 0 | 0 | 0 | 4 | 3 | 0 | 0 | 0 |
|  | (25.0) | (0.0) | (0.0) | (0.0) | (0.0) | (15.4) | (11.5) | (0.0) | (0.0) | (0.0) |
| Skin and Subcutaneous Tissue Disorders | 13 | 0 | 0 | 0 | 0 | 8 | 2 | 1 | 0 | 0 |
|  | (46.4) | (0.0) | (0.0) | (0.0) | (0.0) | (30.8) | (7.7) | (3.8) | (0.0) | (0.0) |
| Vascular Disorders | 2 | 3 | 0 | 0 | 0 | 3 | 1 | 0 | 0 | 0 |
|  | (7.1) | (10.7) | (0.0) | (0.0) | (0.0) | (11.5) | (3.8) | (0.0) | (0.0) | (0.0) |
